# Impact of Parkinson’s disease on the efficiency of masticatory cycles: Electromyographic analysis

**DOI:** 10.4317/medoral.22841

**Published:** 2019-04-24

**Authors:** Nayara da Silva, Edson Verri, Marcelo Palinkas, Jaime Hallak, Simone Regalo, Selma Siéssere

**Affiliations:** 1 DDS, Department Biomechanics, Medicine and Locomotive Apparatus Rehabilitation, Ribeirão Preto Medical , University of São Paulo, São Paulo, Brazil; 2 DDS, PhD, Professor. Department of the Physiotherapy, Claretiano Centro Universitario de Batatais, São Paulo, Brazil; 3 DDS, PhD, Professor. Department of Morphology, Physiology and Basic Pathology, School of Dentistry of Ribeirão Preto, University of São Paulo; Faculty Anhanguera, Ribeirão Preto and National Institute and Technology - Translational Medicine (INCT.TM), São Paulo, Brazil; 4 DDS, PhD, Professor. Department of Neurosciences and Behavioral Sciences of Ribeirão Preto Medical School, University of São Paulo and Coordinator of National Institute and Technology - Translational Medicine (INCT.TM), São Paulo, Brazil; 5 DDS, PhD, Professor. Department of Morphology, Physiology and Basic Pathology, School of Dentistry of Ribeirão Preto, University of São Paulo and National Institute and Technology - Translational Medicine (INCT.TM), São Paulo, Brazil; 6 DDS, PhD, Associated Professor. Department of Morphology, Physiology and Basic Pathology, School of Dentistry of Ribeirão Preto, University of São Paulo and National Institute and Technology - Translational Medicine (INCT.TM), São Paulo, Brazil

## Abstract

**Background:**

This study evaluated the efficiency of masticatory cycles by means of the linear envelope of the electromyographic signal of the masseter and temporalis muscles in individuals with Parkinson’s disease.

**Material and Methods:**

Twenty-four individuals were assigned into two groups: with Parkinson’s disease, average ± SD 66.1 ± 3.3 years (n = 12) and without the disease, average ± SD: 65.8 ± 3.0 years (n = 12). The MyoSystem-I P84 electromyograph was used to analyze the activity of masticatory cycles through the linear envelope integral in habitual mastication of peanuts and raisins and non-habitual mastication of Parafilm M®.

**Results:**

There was statistically significant difference (*P* ≤ 0.05) between individuals with Parkinson’s disease and without the disease in non-habitual mastication of Parafilm M®, in the right temporal muscle (*P* = 0.01); habitual mastication of peanuts, in the right temporal muscle (*P* = 0.02), left temporal muscle (*P* = 0.03), and right masseter muscle (*P* = 0.01); and habitual mastication of raisins in the right temporal muscle (*P* = 0.001), left temporal muscle (*P*= 0.001), right masseter muscle (*P*= 0.001) and left masseter muscle (*P*= 0.03).

**Conclusions:**

These results suggest that Parkinson’s disease interferes in the electromyographic activity of the masticatory cycles by reducing muscular efficiency.

** Key words:**Parkinson’s Disease, electromyography, masticatory efficiency, masseter muscle, temporal muscle.

## Introduction

Parkinson’s disease is a chronic degenerative and progressive disease that produces changes in the central nervous system. These changes involve the basal nuclei, specifically the striatum, which is composed of the caudate nucleus and putamen. In addition, this disease leads to the death of dopaminergic neurons in the substantia nigra (1).

The elderly population has increased considerably due to an increase in life expectancy. Therefore, chronic-degenerative diseases have become more common, thus forming a new epidemiological profile (2). Parkinson’s disease commonly affects individuals older than 50 years of age, although it can be diagnosed in young adults and adolescents (3). Approximately 2% of the world’s population over 65 years of age is affected by the disease, which is considered the second most common senile disease. In addition, Parkinson’s disease is equally prevalent across ethnic groups and social classes and has a low prevalence in males (4).

The physiological changes that Parkinson’s disease triggers can compromise functions and balance and promote alterations in the stomatognathic system (5). This complex anatomical system has structures specialized for specific functions, and any alteration due to degenerative diseases can produce a functional imbalance (6).

Neurodegenerative diseases cause motor alterations that affect the musculoskeletal system (7). Previous studies have reported that over 50% of the individuals diagnosed with Parkinson’s disease exhibit eating disorders and dysfunction in the masticatory process (8); however, there is little information in the literature about the impact of this disease on the function of the masticatory muscles. This study is necessary to better understand the functional alterations of the stomatognathic system and observe the impact of the disease on the masticatory system. The hypothesis of the study is that Parkinson’s disease negatively influences the performance of the masticatory muscles. The aim was to evaluate the efficiency of masticatory cycles during the chewing of soft and hard foods of Parkinson’s patients compared to individuals without the disease.

## Material and Methods

-Sample

This research was approved by the Committee of Ethics in Research with Humans at the Claretian University Center of Batatais, São Paulo, Brazil (protocol # 61113916.6.0000.5381). All participants signed a free and informed consent form, in accordance with Resolution 466/2012 of the National Health Council.

Individuals with Parkinson’s disease were diagnosed by the neurologist and were recruited from the Department of Neurology, Claretian University Center, Batatais, São Paulo, Brazil.

The Hoehn and Yahr scale was used to determine the degree of impairment in individuals with Parkinson’s disease (9), and the Mini Mental State Examination (MMSE) was employed to evaluate cognitive function (10), whose result was 28.08 points. A trained professional administered these examinations.

A post hoc sample size calculation was conducted considering a level of α=0.05, a power of 100% for the main outcome Parafilm M® chewing (mean of the right temporal muscle, PG = 1.90 [0.34] and CG= 0.98 [0.11]), effect size of 3.64. The minimal sample size obtained was 24 volunteers (12 for each group). Sample size calculation was performed with the G*Power 3.0.10 software. 

A total of 54 individuals with Parkinson’s disease, between 50 and 70 years, were evaluated in this study. Following the exclusion criteria, 12 individuals with Parkinson’s disease (average ± SD 66.1 ± 3.3 years), Angle Class I, contact pattern in maximum intercuspal position with tooth to two tooth occlusion and presence of all permanent teeth (except third molars) were selected (grade I and III of the Hoehn and Yahr Scale). The disease-free group (n=12; average ± SD 65.8 ± 3.0 years) was composed of dentate individuals, without temporomandibular dysfunction (RDC/TMD) who were age-, gender-, weight-, and height-matched with individuals in the Parkinson’s disease group.

There were no statistically significant differences between the groups in age (*P* = 0.80), weight in kg (with Parkinson’s disease: 69.08 ± 3.87; disease-free: 67.75 ± 2.70, *P* = 0.34), or height in cm (with Parkinson’s disease: 166 ± 0.08; disease-free: 168 ± 0.08, *P* = 0.61).

The exclusion criteria involved the temporomandibular dysfunction (RDC/TMD, n=08); absence of complete dentition (n=9), presence of ulcers and cutaneous hypersensitivity, a cognitive deficit (MMSE score below 24, n=03), neurological and systemic (decompensated) pathologies associated with the disease, stage IV and V of Hoehn and Yah disability (n=05), inadequate occlusal conditions (i.e. teeth with periodontal mobility, n=10), and use of anti-inflammatories, analgesics and muscle relaxants that could interfere in neuromuscular physiology (n=7). In addition, it was required that individuals with Parkinson’s disease used the drug levodopa to control their symptoms (12).

-Electromyographic analysis - masticatory efficiency

The electromyographic signals of the masticatory cycles were collected using the MyoSystem-I P84 portable electromyograph (DataHominis, Uberlandia, Minas Gerais, Brazil), with analog bandpass filters for a cutoff frequency of 10-1000 Hz, scanning for sample frequency of 4 kHz, and 12-bit resolution. Silver/silver chloride bipolar surface electrodes (DataHominis Ltda., Model DHT-EASD) with diameter and inter-electrode distance of 10 mm were used.

To reduce impedance, the skin was cleaned with alcohol a few minutes before the surface electrodes were positioned (13). A rectangular stainless steel electrode (3 x 4 cm) (Bio-logic Systems Corp., Mundelein, IL, Chicago) was also used as a reference electrode to reduce noise acquisition, fixed on the right wrist of the individual Surface electrodes were positioned according to the recommendations of Surface EMG for Non-Invasive Assessment of Muscles (SENIAM) (14).

A quiet environment was maintained while recording the electromyographic data of the masticatory cycles through the ensemble average analysis, which consists of using the integrated amplitude values of the linear envelopment of the masticatory cycles. The electromyographic signals were acquired in the clinical condition of the free habitual mastication of food with hard consistency (5g. peanuts) and soft consistency (5g. raisins). The non-habitual mastication was obtained with chewing of an inert material constituted by a sheet of paraffin (Parafilm M®), which was folded (18 × 17 × 4 mm, weight 245 mg) placed on both sides of the dental arches. During the non-habitual mastication, subjects were asked to make a movement of short opening so as to reduce the effects of the change in length × tension in the muscle, in typical dynamic records. The data of all the masticatory cycles were collected in 10 s (15).

The individuals remained seated, feet resting on the ground and palms on the thighs, with an erect neck in order to keep the Frankfurt plane parallel to the ground. The individuals were instructed to remain calm and to keep the inspiratory and expiratory movements well paused (16).

At the beginning of the masticatory process, the initial cycles showed a variation in the pattern of the mandibular movement. Therefore, to calculate the results obtained from the integral of the linear envelope of the masticatory cycles, the initial masticatory cycles were eliminated while the central cycles of the electromyographic were maintained. Three initial masticatory cycles were excluded since, in the initial phase of the masticatory process, the first cycles vary considerably during mandibular movements (15).

-Method Error

The method error of the habitual and non-habitual masticatory efficiency measurements was calculated with the Dahlberg formula using the records of five individuals from two different sessions, with a seven-day intersession interval. There was a small variation in the measurements between the first and second sessions for electromyography (3.74%). Intra-rater reliability was analyzed using the calculation of the intraclass coefficient (ICC). Reliability for electromyographic activity was considered good (ICC = 0.936).

-Statistical Analysis

After obtaining the masticatory efficiency data, a normality test was run, and the data were considered normally distributed. The efficiency of the masticatory cycle was analyzed after calculating the integral of the linear envelope of the normalized electromyographic signal. The data were normalized by dental clenching in maximum voluntary contraction and were then statistically analyzed (Statistical Package for the Social Sciences Version 22.0 for Windows, IBM Inc.; Chicago, IL, USA). A descriptive analysis was run to obtain the mean and standard error for each variable. A Student’s t-test (independent samples), with a significance level of 5% and a 95% confidence interval, was used to determine if there were significant differences between the groups.

## Results

 Table 1 shows the standard electromyographic data for habitual mastication (peanuts and raisins) and non-habitual mastication (Parafilm M®) for the groups. Normalized electromyographic means for the masticatory muscles were higher in the individuals with Parkinson’s disease compared to the individuals without the disease. Specifically, there was a statistically significant difference (*P* ≤ 0.05) for the right temporal muscle (*P* = 0.01) in the mastication of Parafilm M®, the right temporal muscle (*P* = 0.02), the left temporal muscle (*P* = 0.03) and the right masseter muscle (*P* =0.01) in mastication of peanuts, and the right temporal muscle (*P* = 0.001), the left temporal muscle (*P* = 0.001), the right masseter muscle (*P* = 0.001) and the left masseter muscle (*P* = 0.03) in the mastication of raisins.

**Table 1:**
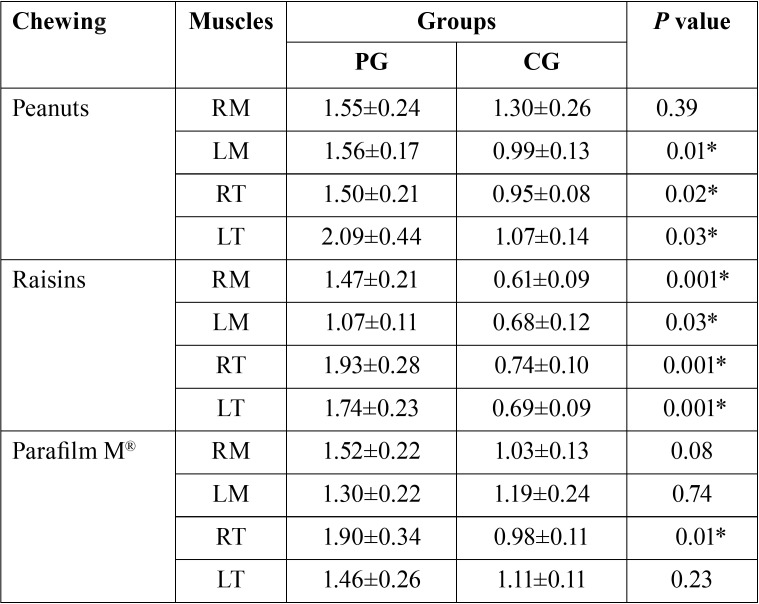
Mean (standard error) and statistical significance (*P* ≤ 0.05)* of the normalized electromyographic data of the right masseter (RM), left masseter (LM), right temporal (RT) and left temporal (LT) muscles for Parkinson’s disease (PG) and without the disease (CG) groups in the habitual and non-habitual chewing.

## Discussion

This study showed that individuals with Parkinson’s disease demonstrated significant changes in masticatory efficiency. The lower masticatory efficiency in people with Parkinson’s disease is relevant, because most of these individuals suffer from considerable involuntary weight loss, masticatory difficulties and malnutrition, mainly due to inadequate food intake (8).

These changes were observed in the non-habitual mastication of Parafilm M® and habitual mastication of soft and consistent food, and in the normalized electromyographic means of the largest masticatory cycles in the individuals with Parkinson’s disease group versus individuals without the disease group. These data are characteristic of dysfunction and a lack in efficiency, and we can conclude that to perform the same function, the energy expenditure was higher in the individuals with Parkinson’s disease group compared to the individuals without the disease group.

The dynamic short-excursion movement of the buccal opening, in order to reduce the effects of changing length and muscular tension, was used to identify non-habitual mastication (17). We observed that the individuals with Parkinson’s disease group demonstrated increased electromyographic activity of the masticatory cycles relative to the individuals without the disease group for all the muscles evaluated.

To perform the masticatory movement, it is necessary to perceive different textures of food, and proprioception is greater when chewing soft foods compared to consistent foods (18). The results of this research suggest that to perform masticatory movements, individuals with Parkinson’s disease recruited a greater number of muscle fibers compared to individuals without the disease. This lead to an increased energy expenditure, and suggested that there is a functional impairment in the individuals with Parkinson’s disease (5).

For dynamic movement to have adequate muscle efficiency, force production is necessary but with less activation of muscle fibers (19). The alteration of the body posture in Parkinson’s disease changes the cervical and mandibular biomechanical relationships in the static and dynamic positions of the stomatognathic system (6).

All the individuals with Parkinson’s disease in this study used Levodopa, a drug that can directly influence musculoskeletal change. This medication is used for long-term response of symptom control, but with continuous use motor deterioration occurs. This deterioration leads to the development of fluctuations, making it difficult to maintain dopaminergic presynaptic terminals, thus reducing the capacity of the skeletal striated musculature to store dopamine (20,21).

As the disease progresses and dopamine storage capacity decreases, the effect of the drug is impaired. Short-term responses begin to appear and slow the motor response (22). This event may explain the musculoskeletal changes that occur in these individuals, and the increased recruitment of motor plaques to perform the masticatory process.

One of the side effects of levodopa is dyskinesia (23), which may alter muscle activity and possibly the masticatory pattern. According to differences between patients and controls may be due to side effects of the treatment, not the disease. It is known that over the years the use of the drug loses the systemic effect and new doses must be adjusted. Further studies should be performed to verify over time the effect of the Levodopa use throughout the skeletal muscle system, including chewing.

Another important factor is that individuals with Parkinson’s disease demonstrate postural alterations that can provide bodily imbalance by modifying the position of the head, which consequently changes the mandibular position (24). This alteration can affect the masticatory pattern, leading to muscular compensation and a decrease in masticatory efficiency (25). In this study, the posture of individuals with Parkinson’s disease was not evaluated.

Patients with a tremor in the face show a deviation in the movement of the mandible, which is a result of the level of dopamine present in the brainstem (26). This situation may also determine the muscular compensations and therefore, functional alterations in the masticatory process.

Among the changes caused by Parkinson’s disease, weight loss is common, often associated with lack of appetite related to the side effects of medications, which contribute to low food intake. These effects may also be associated with lower masticatory efficiency. Therefore, analyzing the results obtained in this research made it possible to observe functional changes in the performance of the stomatognathic system, specifically in the efficiency of the masticatory cycles in individuals with the disease.

Healthcare professionals should take great care when proposing rehabilitative treatments, especially in relation to food intake and nutritional status of individuals with Parkinson’s disease. In addition, these treatments should include multidisciplinary follow-up, which should involve a nutritionist, speech therapist, physiotherapist, dental surgeon, and physician. As the number of individuals with Parkinson’s disease is small, further studies should be performed with a larger sample.

## Conclusions

Based on the results of this study, we suggest that the masticatory cycles of the electromyographic signal for the masseter and temporal muscles in the habitual mastication of soft and consistent food are lower efficient in individuals with Parkinson’s disease when compared to individuals without the disease.
